# SARS-CoV-2 envelope protein triggers depression-like behaviors and dysosmia via TLR2-mediated neuroinflammation in mice

**DOI:** 10.1186/s12974-023-02786-x

**Published:** 2023-05-08

**Authors:** Wenliang Su, Jiahang Ju, Minghui Gu, Xinrui Wang, Shaozhuang Liu, Jiawen Yu, Dongliang Mu

**Affiliations:** 1 Department of Anesthesiology, Peking University First Hospital, Beijing, China; 2 Liangzhu Laboratory, MOE Frontier Science Center for Brain Science and Brain-Machine Integration, State Key Laboratory of Brain-Machine Intelligence, Zhejiang University, Hangzhou, 311121 China; 3 Department of Rehabilitation Medicine, Tongji Hospital, Tongji Medical College, Huazhong University of Science and Technology, Wuhan, Hubei China; 4 Department of Pharmacy, Beijing Chaoyang Hospital, Capital Medical University, Beijing, China; 5 Department of Urology, Shengjing Hospital of China Medical University, Sanhao Street 36, Shenyang, 110004 Liaoning China; 6 Department of Anesthesiology, Peking Union Medical College Hospital, Chinese Academy of Medical Sciences and Peking Union Medical College, Beijing, China

**Keywords:** COVID-19, Depression, Dysosmia, E protein, TLR2

## Abstract

**Background:**

Depression and dysosmia have been regarded as primary neurological symptoms in COVID-19 patients, the mechanism of which remains unclear. Current studies have demonstrated that the SARS-CoV-2 envelope (E) protein is a pro-inflammatory factor sensed by Toll-like receptor 2 (TLR2), suggesting the pathological feature of E protein is independent of viral infection. In this study, we aim to ascertain the role of E protein in depression, dysosmia and associated neuroinflammation in the central nervous system (CNS).

**Methods:**

Depression-like behaviors and olfactory function were observed in both female and male mice receiving intracisternal injection of E protein. Immunohistochemistry was applied in conjunction with RT-PCR to evaluate glial activation, blood–brain barrier status and mediators synthesis in the cortex, hippocampus and olfactory bulb. TLR2 was pharmacologically blocked to determine its role in E protein-related depression-like behaviors and dysosmia in mice.

**Results:**

Intracisternal injection of E protein evoked depression-like behaviors and dysosmia in both female and male mice. Immunohistochemistry suggested that the E protein upregulated IBA1 and GFAP in the cortex, hippocampus and olfactory bulb, while ZO-1 was downregulated. Moreover, IL-1β, TNF-α, IL-6, CCL2, MMP2 and CSF1 were upregulated in both cortex and hippocampus, whereas IL-1β, IL-6 and CCL2 were upregulated in the olfactory bulb. Furtherly, inhibiting microglia, rather than astrocytes, alleviated depression-like behaviors and dysosmia induced by E protein. Finally, RT-PCR and immunohistochemistry suggested that TLR2 was upregulated in the cortex, hippocampus and olfactory bulb, the blocking of which mitigated depression-like behaviors and dysosmia induced by E protein.

**Conclusions:**

Our study demonstrates that envelope protein could directly induce depression-like behaviors, dysosmia, and obvious neuroinflammation in CNS. TLR2 mediated depression-like behaviors and dysosmia induced by envelope protein, which could serve as a promising therapeutic target for neurological manifestation in COVID-19 patients.

**Supplementary Information:**

The online version contains supplementary material available at 10.1186/s12974-023-02786-x.

## Introduction

The COVID-19 pandemic has dramatically impacted the world since the SARS-CoV-2 virus infection outbreak in 2019. Clinical symptoms of COVID-19 could involve several human body systems, apart from the most commonly known respiratory system symptoms. The COVID-19 infection can cause a series of neurological symptoms, including attention disorders, sleep disorders, short-term memory loss, seizures, strokes, headaches, dizziness, smell and taste loss, and neuropsychiatric symptoms, such as anxiety and depression [1–8]. Furthermore, brain MRI imaging of COVID-19 patients showed a decrease in overall brain size, prefrontal brain, and parahippocampal gyrus gray matter thickness. Additionally, changes of tissue damage markers in the primary olfactory cortex regions were more significant compared with the negative control group [9]. Further pathological observation revealed that brain samples from patients with COVID-19 showed replication of SARS-CoV-2 in CNS and infection of glial cells by the virus [10]. However, the relationship between SARS-CoV-2 infection and COVID-19 neurological symptoms remains unclear.

The SARS-CoV-2 virus has four structural proteins: the membrane protein, spike protein, nucleocapsid protein, and envelope protein (E) [11]. The E is a small integral protein composed of only 75 amino acids. However, E involves many processes in the virus's life cycle, such as assembly, budding, envelope formation, and pathogenic mechanism [12]. The E is highly expressed in infected cells during the virus's replication cycle [13]. E plays a vital role in the production and maturation of the virus. Evidence reveals that recombinant coronavirus lacking E showed significantly decreased virus titers and offspring with impaired virus maturation or insufficient production and proliferation [14, 15]. Meanwhile, E, as a multifunctional protein, not only acts as a structural component in the virus capsid and participates in virus assembly, but also acts as a virulent toxin and participates in the pathogenesis of the virus [16, 17].

Previous evidence reported that the E protein of SARS-CoV-2 could bind to the Toll-like receptors (TLRs) and induce pulmonary inflammation [18]. Additionally, the E protein is also necessary for the release of inflammatory cytokines during coronavirus infection. It was found that the E protein could interact with TLR2 receptors and induce the expression of tumor necrosis factor, interferon-γ, interleukin 6, and interleukin 1β in human peripheral blood mononuclear cells. The SARS-CoV-1 strain lacking the E protein was unable to activate the NF-κB signal transduction pathway, resulting in a significant decrease in the production of inflammatory cytokines [19]. Combined the neurological SARS-CoV-2 infection with the essential role of E protein–TLRs interaction in the virus pathogenesis, we hypothesize that the COVID-19 patients’ dysosmia and depression symptoms might be mediated by the interaction of E protein with TLRs of glial cells.

## Methods

### Animals

Wild type C57BL/6 male and female mice (20–25 g; 6–8 weeks), purchased from the National Institutes for Food and Drug Control in China, were used in this study. Animals were housed under a 12-h light/12-h dark cycle with ad libitum access to food and water.

### Sucrose preference test (SPT)

The mice were housed in cages with a two-bottle-choice setting for 48 h (one bottle for water and the other for 1.5% sucrose solution). In addition, the position of the two bottles was switched at 24 h. Water access was then deprived from the tested mice for 24 h. The two-bottle drinking setting was provided again for the tested mice for 2 h, during which the position of the two bottles was switched at 1 h. The ratio of sucrose solution consumed to the total fluid intake was determined as the sucrose preference [20].

### Tail suspension test (TST)

The TST was performed as previously described. Briefly, each mouse was suspended by its tail with a short adhesive tape connected to a load cell that transmitted a signal corresponding to activity. The total test time was 6 min. After setting a low threshold, the duration of immobility was recorded and analyzed by tail suspension software (SOF-821, Med Associates) [20].

### Forced swimming test (FST)

Mice were individually placed in a beaker (height: 19 cm; diameter: 14 cm) containing 14 cm of water (23 ± 2 °C). The total test duration was 6 min. The process was videotaped, and the immobility time in the last 4 min was scored by an experienced observer blinded to the experimental treatment. Floating or only slight movement to maintain balance was considered as immobility [20].

### Olfactory measurement (OM)

On the first day, the mice were trained for 3 min in a cage containing sunflower seeds in four corners. Then, the mice were trained for 3 min again, but sunflower seeds were placed only in one corner. Following the training, the mice were separated from their dam and food was withheld for one day. Three 3-min trials were conducted on day 3. In trials 1 and 2, one sunflower seed was placed in the cage on bedding, each time in a different corner. In trial 3, a seed was buried under the bedding at the center of the cage. The latency to find the food was recorded. If mice could not to find a seed within 3 min, the latency to find food was recorded as 180 s. A shorter latency to find buried sunflower seed indicates better olfaction [21].

### Intracisternal injection

**Fig. 1:**
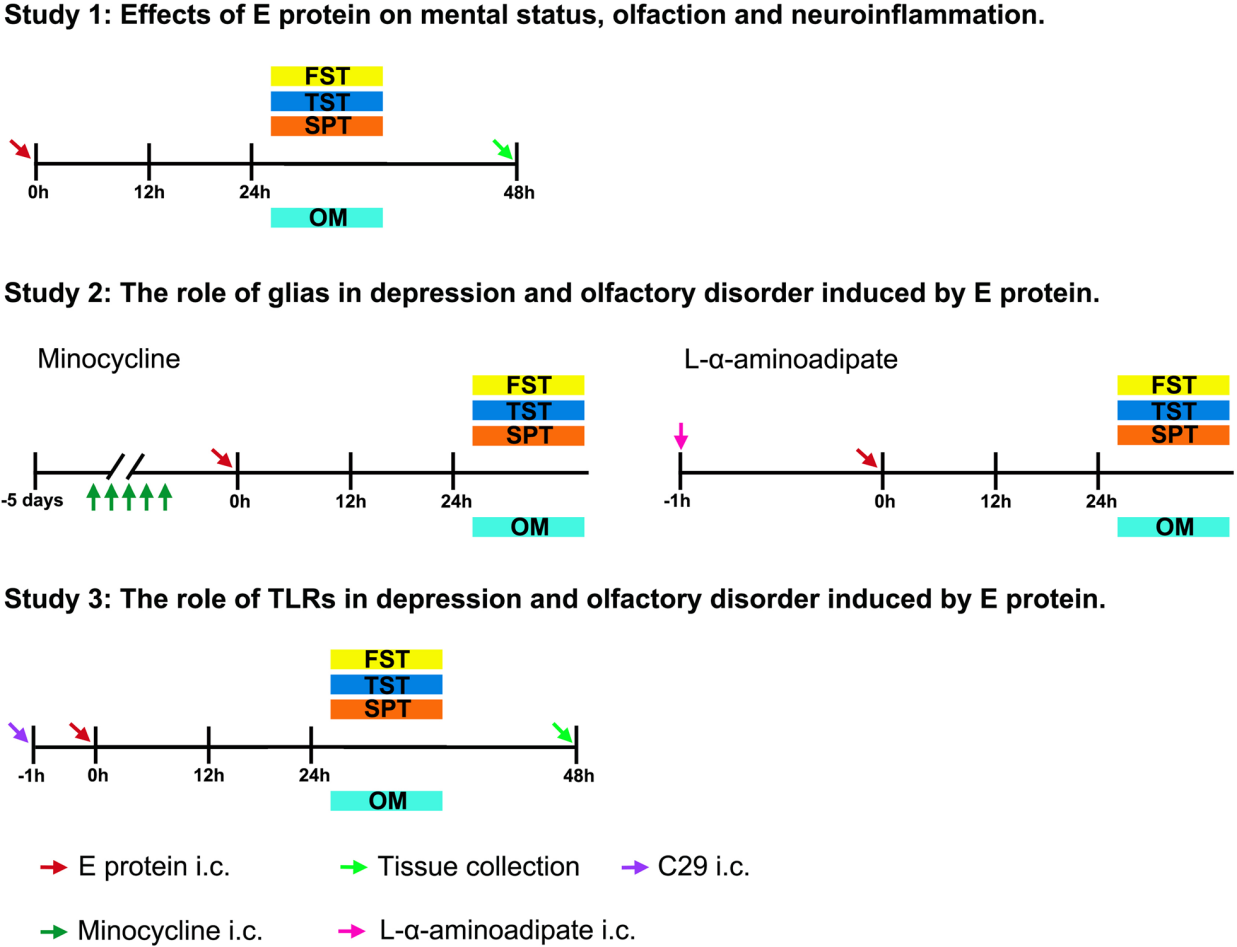
The timeline of E protein injection, drug delivery and behavioral testing

To observe the effects of E protein in CNS, mice received intracisternal injection of E protein. Briefly, E protein (0.2 μg/μl buffered in PBS, 5 μl) (ENN-C5128, Acro Biosystems) or Vehicle (PBS, 5 μl) was slowly injected into the cisterna magna using a specially made length-limited syringe [22–24]. To inhibit microglia and astrocytes, minocycline (a microglial inhibitor, 5 μg in 5 μl PBS, delivered daily for constructive 5 days prior to E protein application) (M9511, sigma) and L-α-aminoadipate (LAA, an astroglial toxin, 50 nmol buffered in 1N HCl and further diluted in PBS, delivered 1 h prior to E protein application) (A7275, sigma) were applied via intracisternal injection [25, 26]. To specifically block TLR2, C29 (a specific TLR2 inhibitor, 50 mg/kg, buffered in PBS containing 10% DMSO) was injected into the cisterna magna via syringe 1 h prior to E protein application. The timeline of drug delivery, behavior test, and tissue collection is depicted in Fig. 1.

### Immunofluorescence

Immunofluorescence (IF) was performed as previously described. Briefly, mice were anesthetized with intraperitoneal sodium pentobarbital injection (40 mg/kg) under aseptic condition and then transcardially perfused with ice-cold PBS followed by ice-cold paraformaldehyde. The whole brain from each tested mouse was collected, post-fixed in 4% paraformaldehyde at 4 °C for 2 h, then dehydrated in 30% sucrose at 4 °C. Then, these brain tissues were embedded in OCT (Tissue-Tek, Japan) and serially sectioned in a cryostat (Leica 2000, Germany) into 30-μm-thick slices. The tissue sections were blocked with 5% donkey serum containing 0.3% Triton X-100 for 1 h, and then primary antibodies were incubated at 4 °C overnight in a wet box. After washing in PBS buffer, secondary antibodies were incubated for 1 h at room temperature (primary antibodies list: ZO-1, ab 221547, abcam, 1:200; IBA1, ab5076, abcam, 1:1000; GFAP, ab53554, abcam, 1:1000; TLR2, ab209216, abcam, 1:200. Secondary antibody list: Donkey anti-Rabbit 488, A32790, Invitrogen, 1:500; Donkey anti-Mouse 488, A-21202, Invitrogen, 1:500; Donkey anti-Goat 488, ab150129, abcam, 1:500). The slides were then coverslipped by Mounting Medium solution containing DAPI (ZSJB-Bio, Beijing, China). Images were captured by a laser confocal microscopic imaging system under the same settings (TCS-SP8 STED 3X, Leica, Germany). The quantification for IF staining referred to previous studies [27]. At least 10 sections from 3 randomly selected mice in each group were examined. The positive area of IBA1, GFAP, ZO-1 and TLR2 staining was measured with Image J.

### Quantitative RT-PCR

Total RNAs from the cortex, hippocampus, and olfactory bulb were extracted using Trizol reagent (CW-bio, Beijing, China) and then reverse transcribed by RT Master Mix according to the instruction (Takara, Japan). Finally, qRT-PCR was performed using a CFX96™ Real-Time PCR Detection System (Bio-Rad, California, USA) with TB Green Premix Ex Taq (Takara, Japan). The primers used are listed in Additional file 4: Table S1.

### Statistical analysis

Data values were presented as means with standard errors (mean ± SEM). Statistical analyses were performed by Graph Pad software. The Shapiro–Wilk test was applied to determine the normality for the parametric test. The Student’s *t*-test was used to determine the statistical difference between two groups. The criterion for statistical significance was a value of *p* < 0.05.

## Results

### E protein induced depression-like behaviors and dysosmia in female and male mice

**Fig. 2:**
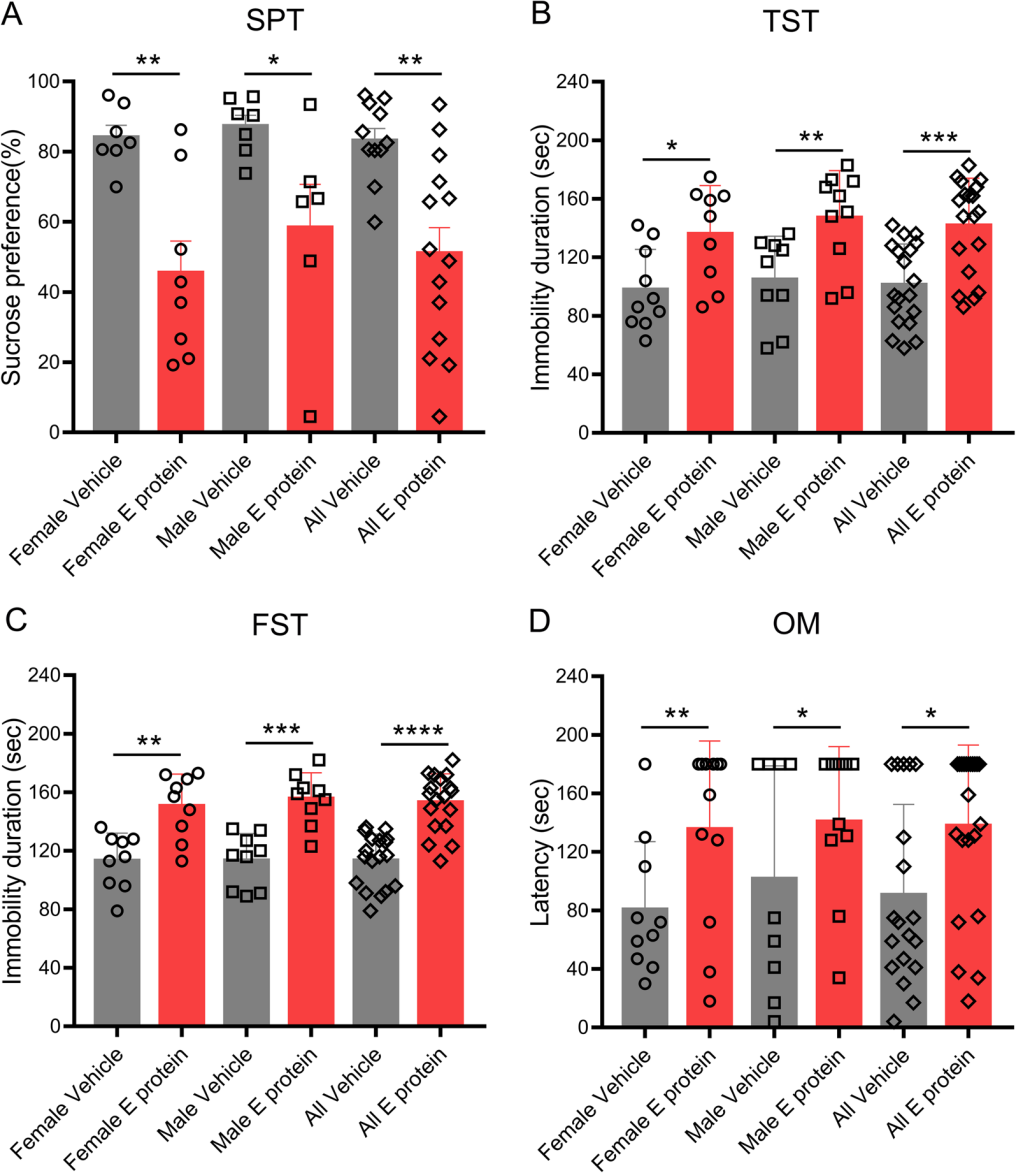
The SPT, TST, FST, and OM for mice receiving intracisternal injection of E protein. **A** The percentage of sucrose water consumption for female and male mice receiving Vehicle or E protein injection. **B.** The immobility duration in TST test for female and male mice receiving Vehicle or E protein intracisternal injection. **C** The immobility duration in FST test for female and male mice receiving Vehicle or E protein intracisternal injection. **D** The latency time for discovering the sunflower seed in the OM test. *n* ≥ 6 mice per group; *p < 0.05, **p < 0.01, ***p < 0.001, ****p < 0.0001; Student’s *t* test

To identify the neuropathological effects of E protein, we applied intracisternal injection of E protein and evaluated the mental and olfactory status. The SPT, TST, and FST tests indicated that E protein in the CNS significantly induced depression-like behaviors in both female and male mice (Fig. 2A–C). Furthermore, the OM test showed that E protein increased the time to find the target food in both female and male mice, suggesting that E protein evoked olfactory impairment (Fig. 2D). Collectively, we observed typical depression-like behaviors and olfactory disturbances in COVID-19 patients as a result of E protein delivery to the CNS.

### E protein triggered neuroinflammation and blood–brain barrier damage in cortex, hippocampus and olfactory bulb

**Fig. 3:**
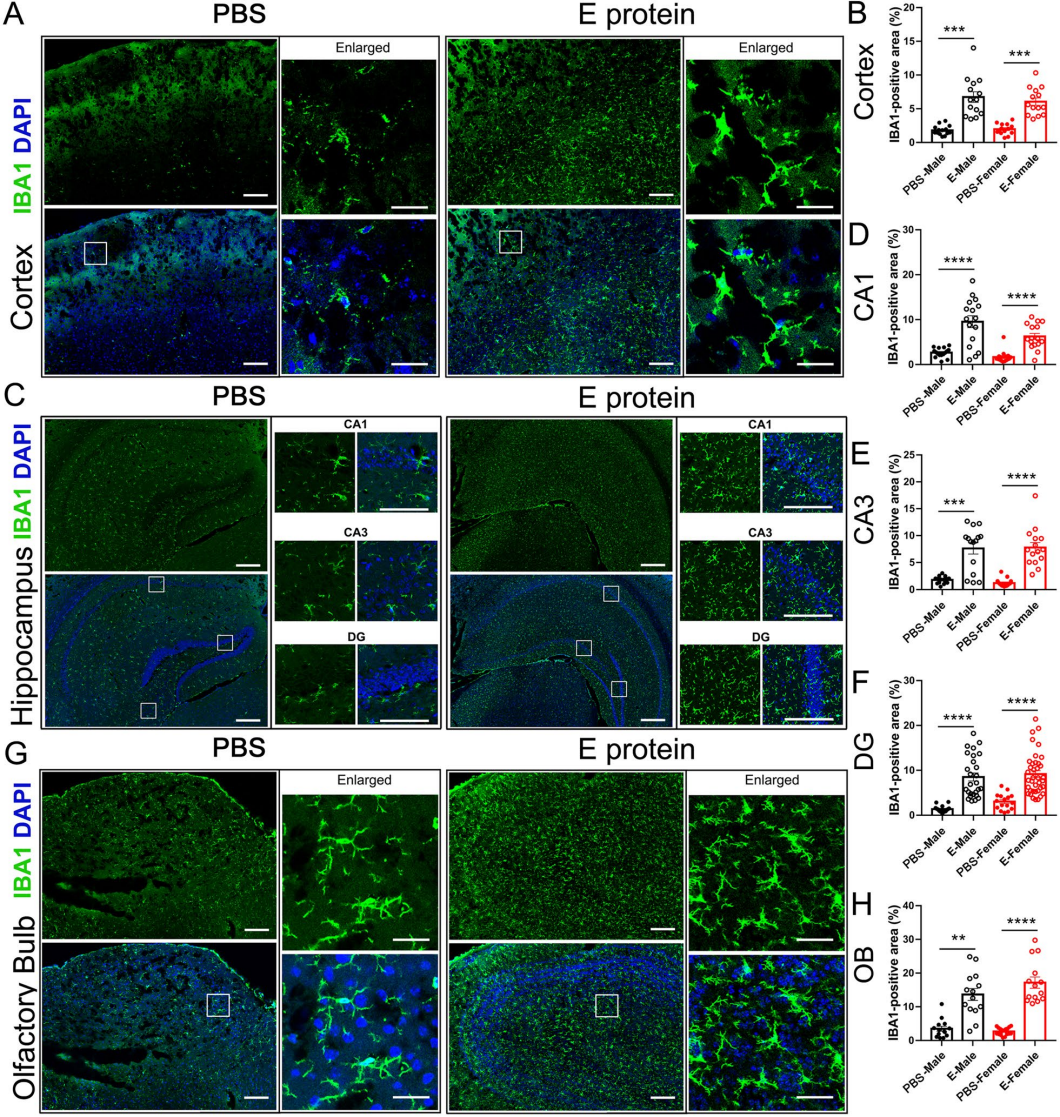
Intracisternal injection of E protein upregulated IBA1 in the cortex, hippocampus and olfactory bulb. **A** Representative images of IBA1 expression in the cortex from mice receiving Vehicle or E protein injection. **B** Fluorescence area analysis showed that E protein significantly upregulated IBA1 expression in the cortex. **C** Representative images of IBA1 expression in the hippocampus (CA1, CA3 and DG regions) from mice receiving Vehicle or E protein injection. **D–F** Fluorescence area analysis showed that E protein significantly increased IBA1 expression in the CA1, CA3, and DG regions of hippocampus. **G** Representative images of IBA1 expression in the olfactory bulb from mice receiving Vehicle or E protein injection. **H** Fluorescence area analysis showed that E protein significantly upregulated IBA1 expression in the olfactory bulb. Scale bar: 50 μm. *n* ≥ 6 per group; *p < 0.05, **p < 0.01, ***p < 0.001, ****p < 0.0001; Student’s *t* test

**Fig. 4:**
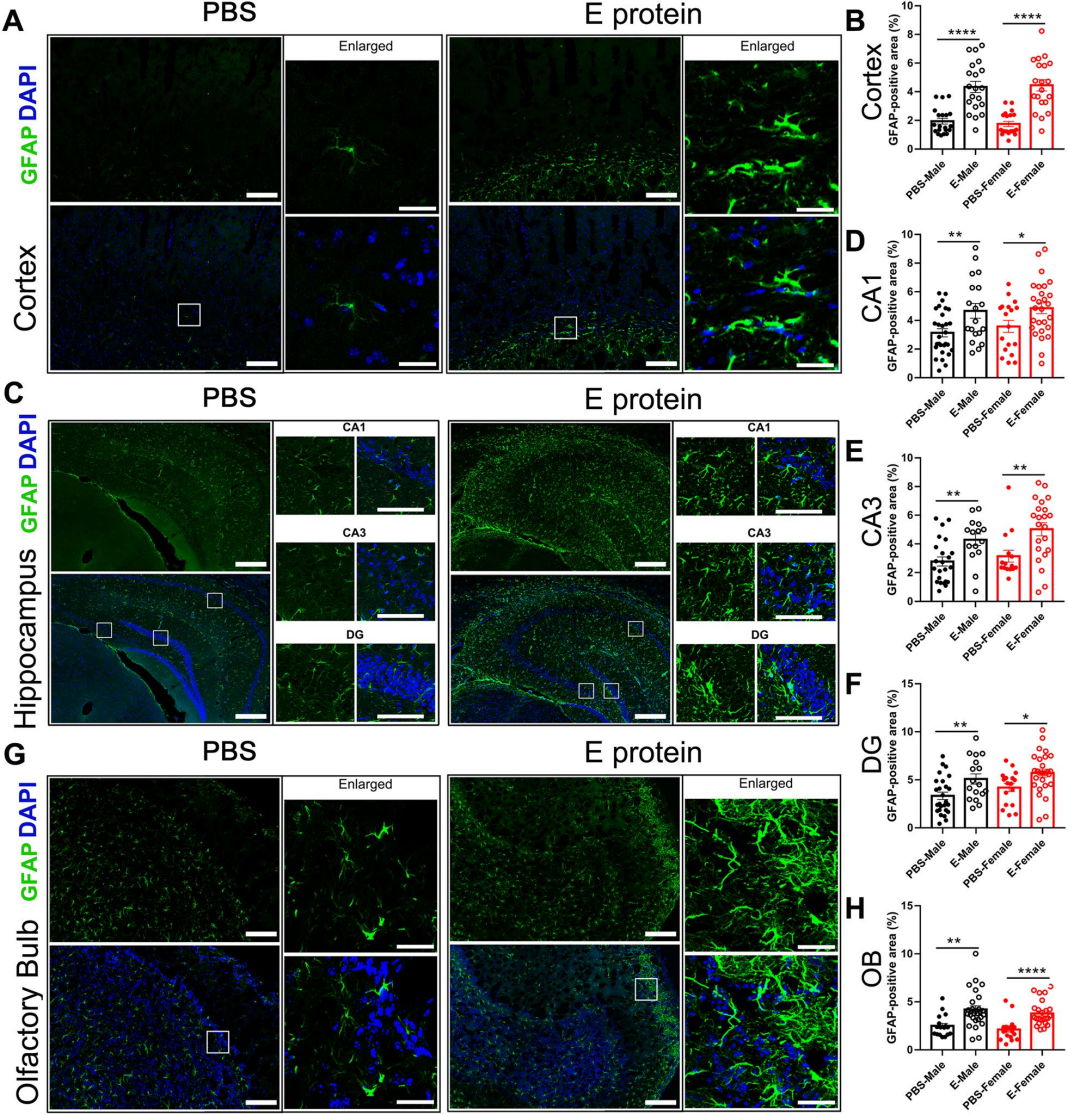
Intracisternal injection of E protein upregulated GFAP in the cortex, hippocampus and olfactory bulb. **A** Representative images of GFAP expression in the cortex from mice receiving Vehicle or E protein injection. **B** Fluorescence area analysis showed that E protein significantly upregulated GFAP expression in the cortex. **C** Representative images of GFAP expression in the hippocampus (CA1, CA3 and DG regions) from mice receiving Vehicle or E protein injection. **D–F** Fluorescence area analysis showed that E protein significantly upregulated GFAP expression in the CA1, CA3 and DG regions of the hippocampus. **G** Representative images of GFAP expression in the olfactory bulb from mice receiving Vehicle or E protein injection. **H** Fluorescence area analysis showed that E protein significantly upregulated GFAP expression in the olfactory bulb. Scale bar: 50 μm. *n* ≥ 6 per group; *p < 0.05, **p < 0.01, ****p < 0.0001; Student’s *t* test

**Fig. 5:**
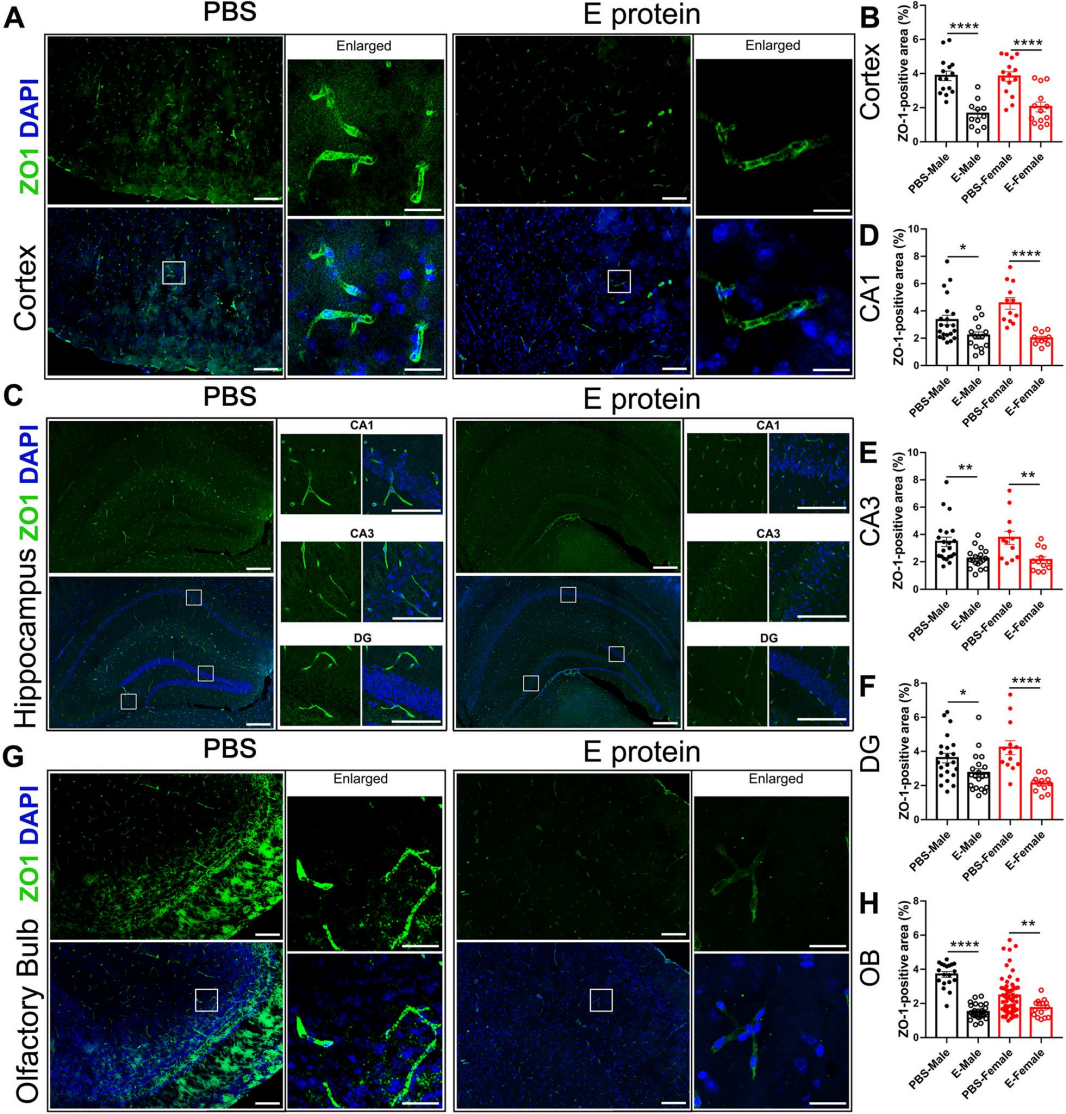
Intracisternal injection of E protein downregulated ZO-1 in the cortex, hippocampus and olfactory bulb. **A** Representative images of ZO-1 expression in the cortex from mice receiving Vehicle or E protein injection. **B** Fluorescence area analysis showed that E protein significantly downregulated ZO-1 expression in the cortex. **C** Representative images of ZO-1 expression in the hippocampus (CA1, CA3 and DG regions) from mice receiving Vehicle or E protein injection. **D–F** Fluorescence area analysis showed that E protein significantly downregulated ZO-1 expression in the CA1, CA3, and DG regions of the hippocampus. **G** Representative images of ZO-1 expression in the olfactory bulb from mice receiving Vehicle or E protein injection. **H** Fluorescence area analysis showed that E protein significantly downregulated ZO-1 expression in the olfactory bulb. Scale bar: 50 μm. *n* ≥ 6 per group; *p < 0.05, **p < 0.01, ****p < 0.0001; Student’s *t* test

**Fig. 6:**
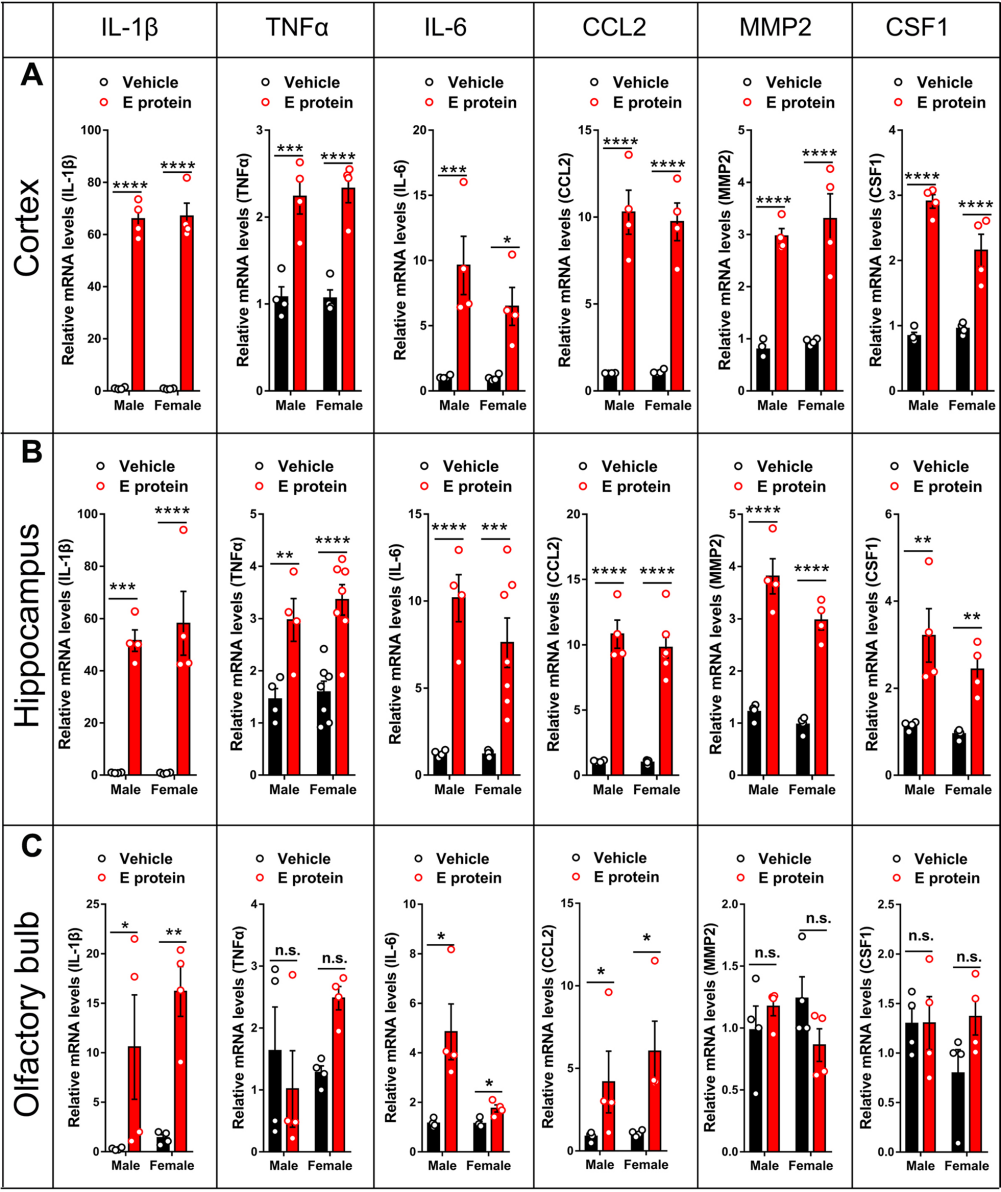
Intracisternal injection of E protein induced neuroinflammatory mediators in the cortex, hippocampus and olfactory bulb. **A** qRT-PCR analysis showed that intracisternal injection of E protein upregulated IL-1β, TNF-α, IL-6, CCL2, MMP2 and CSF1 in the cortex. **B** qRT-PCR analysis showed that intracisternal injection of E protein upregulated IL-1β, TNF-α, IL-6, CCL2, MMP2 and CSF1 in the hippocampus. **C** qRT-PCR analysis showed that intracisternal injection of E protein upregulated IL-1β, IL-6, and CCL2 in the olfactory bulb. n = 4; *p < 0.05, **p < 0.01, ***p < 0.001, ****p < 0.0001; n.s., no significance; Student’s *t* test

Neuroinflammation has been regarded as a key cause of depressive and olfactory disorders, so we further systemically assessed the neuroinflammatory status in the cortex, hippocampus and olfactory bulb regions. IF staining for the microglia marker IBA1 suggested significant microglia activation induced by E protein in the cortex (Fig. 3A, B), hippocampus (CA1, CA3 and DG regions) (Fig. 3C–F), and olfactory bulb (Fig. 3G, H) in both male and female mice. Meanwhile, GFAP, the astrocytes marker, was also upregulated by E protein in the cortex (Fig. 4A, B), hippocampus (CA1, CA3 and DG regions) (Fig. 4C–F), and olfactory bulb (Fig. 4G, H). These results indicated that E protein triggered significant glial activation in the cortex, hippocampus and olfactory bulb regions. The destruction of the blood–brain barrier (BBB) plays a critical role in the formation of neuroinflammation; therefore, we also tested the expression of tight junction marker ZO-1 to evaluate BBB status. The IF staining showed a significant decrease of ZO-1 expression in the cortex (Fig. 5A, B), hippocampus (CA1, CA3 and DG regions) (Fig. 5C–F), and olfactory bulb (Fig. 5G, H) induced by E protein injection in both female and male mice. Further, we screened the typical neuroinflammatory mediators in the relative brain areas. The RT-PCR results showed that the mRNA expression levels of IL-1β, TNF-α, IL-6, CCL2, MMP2 and CSF1 were upregulated in the cortex and hippocampus. Meanwhile, the mRNA levels of IL-1β, IL-6 and CCL2 were increased in the olfactory blob (Fig. 6). Together, the above results suggested the occurrence of neuroinflammation induced by E protein in brain regions related to depression-like behaviors and dysosmia.

### Inhibiting microglia mitigated depression-like behaviors and dysosmia induced by E protein

**Fig. 7:**
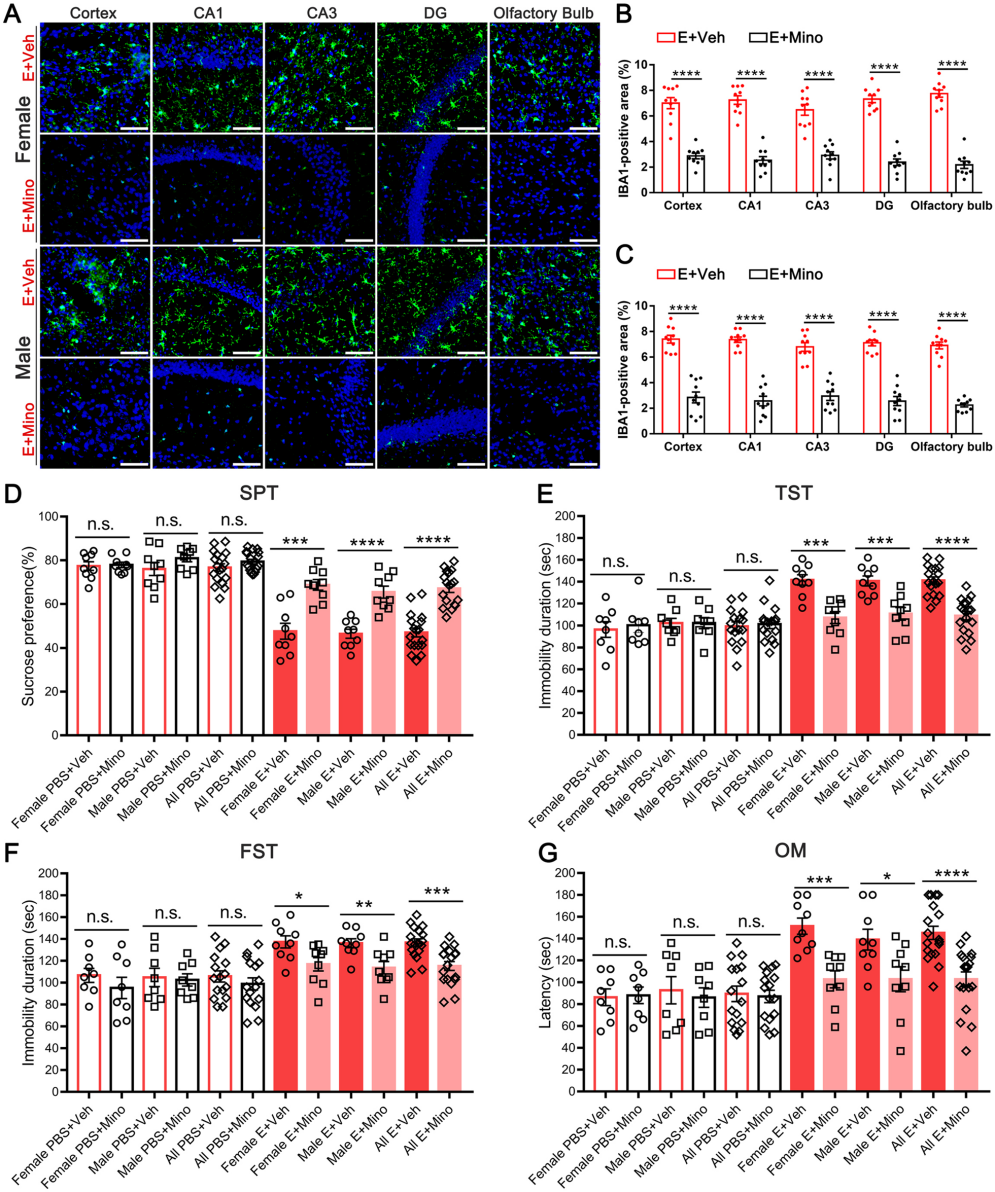
Inhibiting microglia alleviated depression-like behaviors and dysosmia induced by E protein. **A** Representative images of IBA1 expression in the cortex, hippocampus regions, and olfactory bulb from E protein-treated mice receiving Vehicle or minocycline injection.** B, C** Fluorescence area analysis indicated that minocycline significantly downregulated IBA1 expression in the cortex, hippocampus (CA1, CA3, and DG regions), and olfactory bulb (Scale bar: 50 μm). **D** The percentage of sucrose water consumption for PBS- or E protein-treated female and male mice receiving Vehicle or minocycline injection. **E** The immobility duration in TST test for PBS- or E protein-treated female and male mice receiving Vehicle or minocycline injection. **F** The immobility duration in FST test for PBS- or E protein-treated female and male mice receiving Vehicle or minocycline injection. **G** The latency time for discovering the sunflower seed in the olfactory measurement for PBS- or E protein-treated female and male mice receiving Vehicle or minocycline injection. *n* ≥ 6 mice per group; *p < 0.05, **p < 0.01, ***p < 0.001, ****p < 0.0001; n.s., no significance; Student’s *t*-test

Both microglia and astrocytes are essential components in neuroinflammation and neuropathological process. To further investigate the contribution of microglia and astrocytes in E-induced depression-like behaviors and dysosmia symptoms, we used minocycline and LAA to inhibit microglia and astrocytes, respectively, before E protein administration. Immunofluorescence staining suggested that minocycline significantly downregulated the expression of IBA-1 in the cortex, hippocampus, and olfactory bulb (Fig. 7A–C). Similarly, the GFAP molecule expression was inhibited by LAA (Additional file 1: Figure S1A-C). The SPT, TST, and FST tests showed that microglia inhibition by minocycline could successfully alleviate E-induced depression-like behaviors and olfactory disorder in mice (Fig. 7D–G). However, the astrocytes inhibition by LAA failed to recover the depression-like behaviors and olfactory disorder in mice at our observed time point (Additional file 1: Figure S1D-G). Furtherly, minocycline blocked the pro-inflammatory cytokines production induced by E protein (Additional file 2: Figure S2), which might serve as the key source of depressive behavior and dysosmia. Taken together, these results indicated that it was microglia rather than astrocytes that mediated E-induced depression-like behaviors and dysosmia.

### E protein upregulated TLRs in cortex, hippocampus and olfactory bulb

**Fig. 8:**
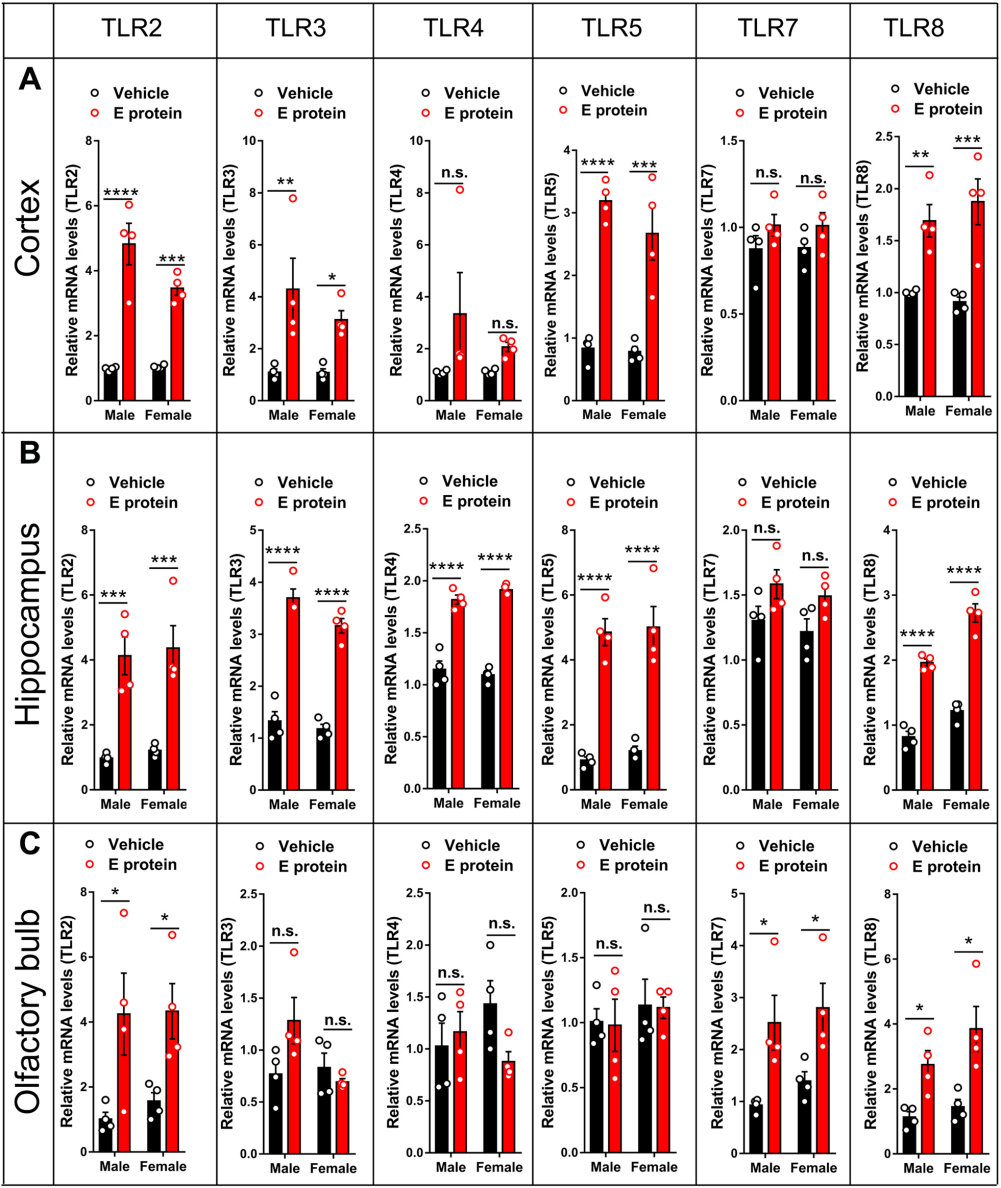
Intracisternal injection of E protein upregulated TLRs in the cortex, hippocampus and olfactory bulb. **A** qRT-PCR analysis showed that intracisternal injection of E protein upregulated TLR2, TLR3, TLR5 and TLR8 in the cortex. **B** qRT-PCR analysis indicated that intracisternal injection of E protein upregulated TLR2, TLR3, TLR4, TLR5, and TLR8 in the hippocampus. **C** qRT-PCR analysis showed that intracisternal injection of E protein upregulated TLR2, TLR7, and TLR8 in the olfactory bulb. *n* = 4; *p < 0.05, **p < 0.01, ***p < 0.001, ****p < 0.0001; n.s., no significance; Student’s *t* test

As an exogenous protein, E protein may be recognized by Toll-like receptors, a group of classic pattern recognition receptors. To identify the possible interaction between TLRs and E protein, we investigated the TLRs mRNA expression in associated brain regions. The RT-PCR results showed that TLR2, TLR3, TLR5, and TLR8 mRNA expression were upregulated in the cortex (Fig. 8A). Additionally, the E protein injection also significantly upregulated TLR2, TLR3, TLR4, TLR5 and TLR8 in the hippocampus (Fig. 8B). In the olfactory bulb, the TLR2, TLR7 and TLR8 mRNA expression levels were upregulated by E protein injection (Fig. 8C). These results indicated that the E protein might take effect via activating the TLRs-dependent signaling pathway. Given that TLR2 was upregulated among three brain regions and its crucial role in microglia function, we further investigated the distribution and role of TLR2 in E-related depression-like behaviors and dysosmia.

### Blocking TLR2 alleviated depression-like behaviors and dysosmia induced by E protein

**Fig. 9:**
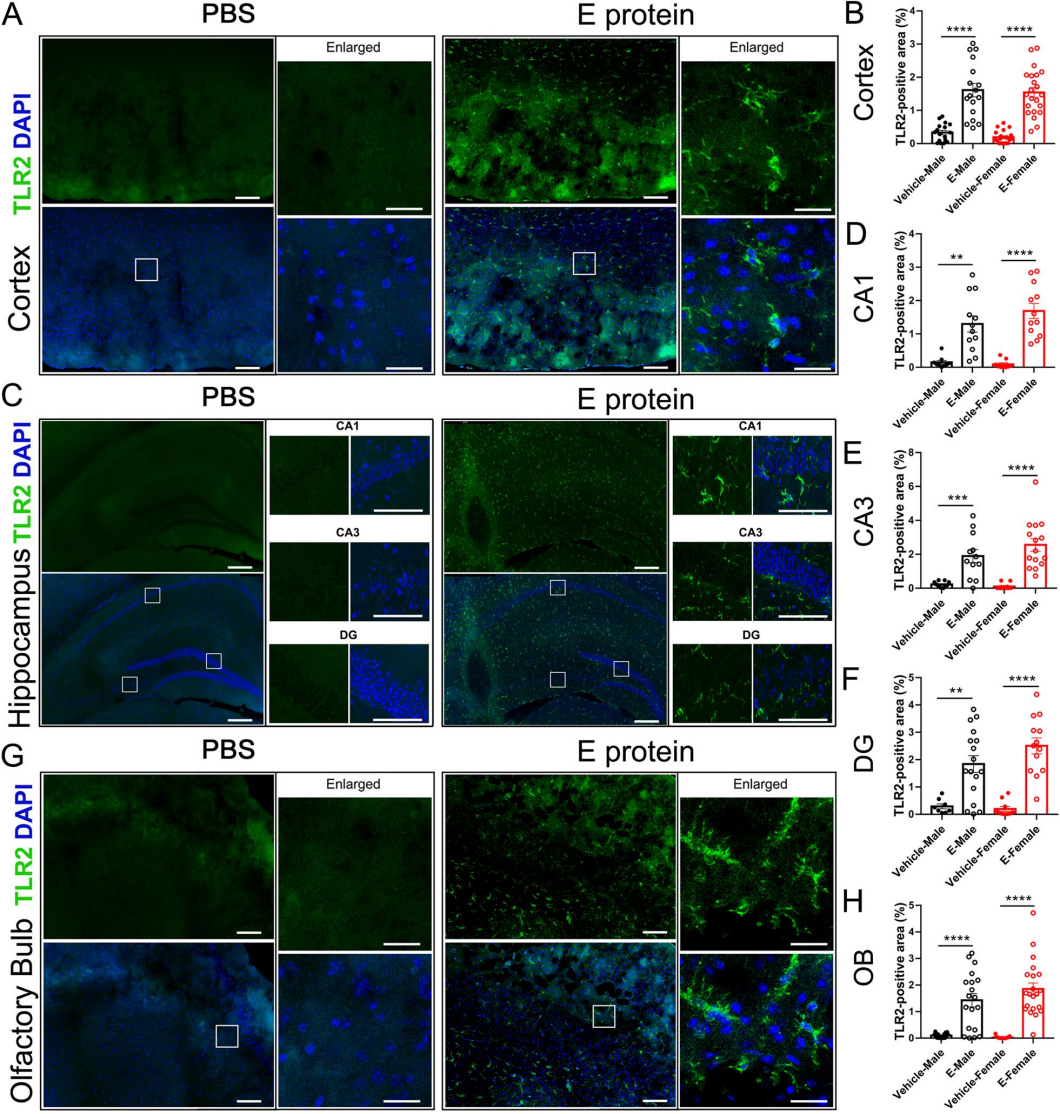
Intracisternal injection of E protein upregulated TLR2 in the cortex, hippocampus and olfactory bulb. **A** Representative images of TLR2 expression in the cortex from mice receiving Vehicle or E protein injection. **B** Fluorescence area analysis showed that E protein significantly upregulated TLR2 expression in the cortex. **C** Representative images of TLR2 expression in the hippocampus (CA1, CA3 and DG regions) from mice receiving Vehicle or E protein injection. **D–F** Fluorescence area analysis showed that E protein significantly upregulated TLR2 expression in CA1, CA3 and DG regions of the hippocampus. **G** Representative images of TLR2 expression in the olfactory bulb from mice receiving Vehicle or E protein injection. **H** Fluorescence area analysis showed that E protein significantly upregulated TLR2 expression in the olfactory bulb. Scale bar: 50 μm. *n* ≥ 6 per group; **p < 0.01, ***p < 0.001, ****p < 0.0001; Student’s *t* test

**Fig. 10:**
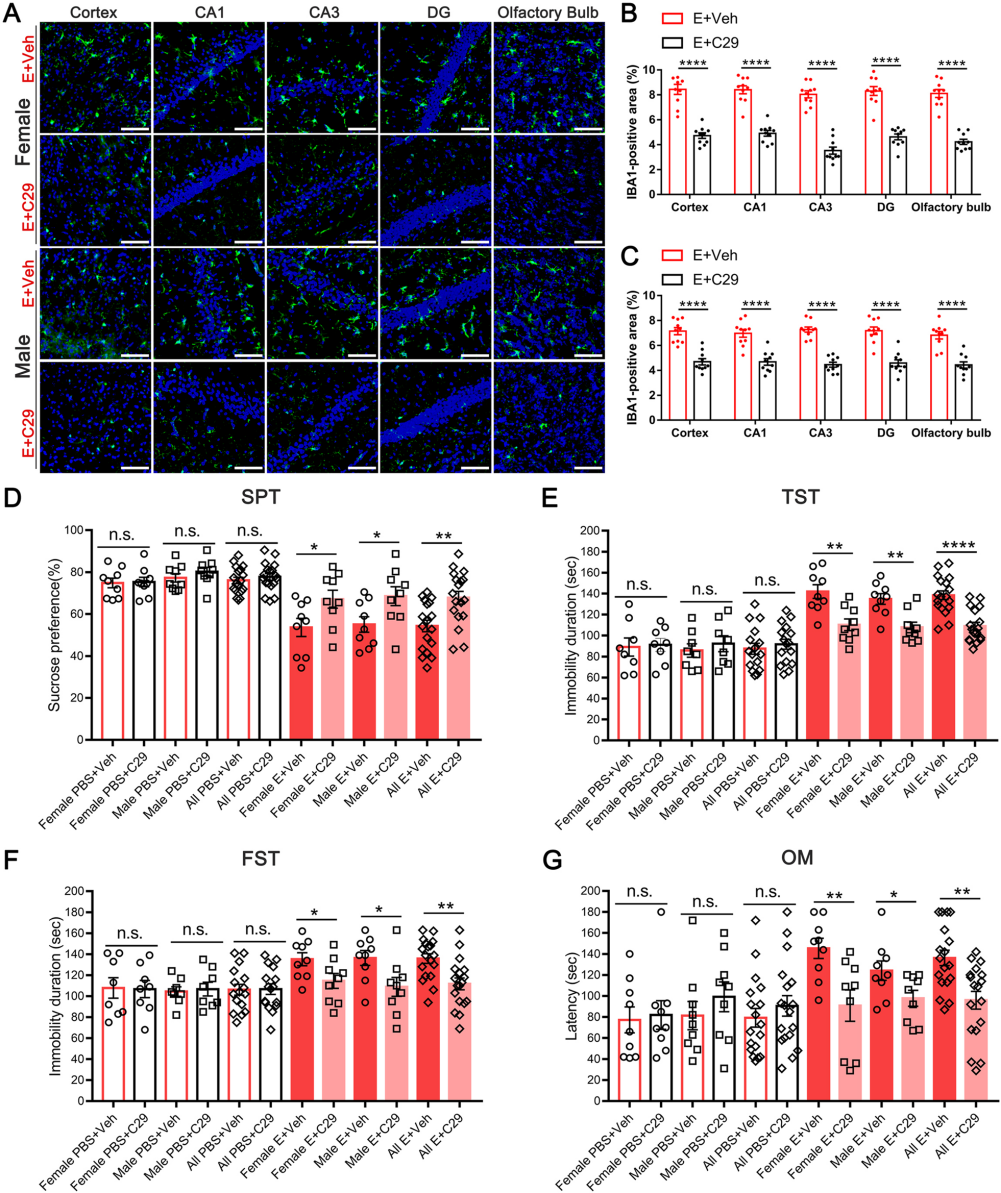
Blocking TLR2 alleviated depression-like behaviors and dysosmia induced by E protein. **A** Representative images of IBA1 expression in the cortex, hippocampus regions, and olfactory bulb from E protein-treated mice receiving Vehicle or C29 injection (scale bar: 50 μm). **B, C** Fluorescence area analysis showed that C29 significantly downregulated IBA1 expression in the cortex, hippocampus (CA1, CA3, and DG regions), and olfactory bulb. **D** The percentage of sucrose water consumption for PBS- or E protein-treated female and male mice receiving Vehicle or C29 injection. **E** The immobility duration in TST test for PBS- or E protein-treated female and male mice receiving Vehicle or C29 injection. **F** The immobility duration in FST test for PBS- or E protein-treated female and male mice receiving Vehicle or C29 injection. **G** The latency time for discovering the sunflower seed in olfactory measurement in PBS- or E protein-treated female and male mice receiving Vehicle or C29 injection. *n* ≥ 6 per group; *p < 0.05, **p < 0.01, ****p < 0.0001; n.s., no significance; Student’s *t*-test

We hypothesized that the E protein triggered neuroinflammation and evoked depression-like behaviors and dysosmia symptoms via TLR2 in CNS. Firstly, IF staining of TLR2 suggested that E protein application to the CNS significantly upregulated TLR2 expression in the cortex (Fig. 9A, B), hippocampus (CA1, CA3 and DG regions) (Fig. 9C–F), and olfactory bulb (Fig. 9G, H). Secondly, we pharmacologically blocked TLR2 using C29a, a specific TLR2 blocker. Simultaneously, the activation of microglia (Fig. 10A–C) and the synthesis of pro-inflammatory cytokines (Additional file 3: Figure S3) were inhibited by C29. The SPT, TST, and FST tests indicated the depression-like behaviors induced by E protein were alleviated by C29 (Fig. 10D–F). Additionally, the shortened latency time in the OM test suggested that the olfactory disorder in mice was also relieved by C29 (Fig. 10G). Overall, these results revealed that blocking TLR2 could alleviate E-induced dysosmia and depression-like behaviors.

## Discussion

Patients with COVID-19 suffer from various neurological and psychiatric symptoms such as dysosmia and depression [28, 29]. The cellular and molecular mechanisms are still elusive, though. The recent discovery provides evidence in support of the notion that the SARS-CoV-2 envelope protein may cause a neuroinflammatory response separate from viral infection [18, 30]. Given these pro-inflammatory properties of the E protein and that neuroinflammation often leads to olfactory dysfunction and mood disorder, we examined the ability of the E protein to trigger inflammatory responses in CNS in vivo and the behavioral sequelae of that response. We demonstrated that intracisternal injection of E protein led to depression-like behaviors and dysosmia as well as neuroinflammation in CNS of mice. Our results suggest that several neurological and psychiatric disorders observed in COVID-19 patients can be mediated by the E protein during SARS-CoV-2 infection.

Hyperactivation of the neuroimmune system is closely related to mood disorders and olfactory dysfunction. There is mounting evidence that microglia play an etiological role in this process [31, 32]. Depression is considered a microglia-associated disorder. Suicidal people and depressive patients exhibit notably elevated microglia activation [33, 34]. Several depression-related brain regions have shown sustained microglial activation exhibiting high amounts of pro-inflammatory cytokines [35–37]. Microglial cells are highly concentrated in hippocampus, particularly in the CA1 region, and activation of these cells has been linked to the pathophysiology of major depressive disorder [38]. Depressive-like behaviors were reduced by inhibiting microglial activation and neuroinflammation [39]. Some clinical antidepressants, such as nonsteroidal anti-inflammatory drugs, appear to reduce the symptoms of depression by preventing the activation of microglia [40–42]. In addition, CNS inflammation is one of the etiologies of olfactory disorders [43]. Olfaction is a crucial sense controlled by the olfactory epithelium's perception of odor molecules, which is then transported to the olfactory bulb via olfactory nerves and processed in the brain [44]. There are lots of microglia in the olfactory bulb. Microgliosis is frequently observed in the olfactory bulb of olfactory dysfunction animals [45, 46]. Olfactory impairments may be caused by the microglial reaction in the olfactory bulb which leads to the loss of neuroblasts [47]. As we observed, the inhibition of microglia effectively suppressed the inflammatory responses in associated brain regions (Additional file 2: Figure S2) and alleviated the depression-like behaviors and olfactory dysfunction induced by the E protein.

The E protein induces potent inflammatory responses as a virulence factor [48]. Microglia and astrocytes were activated by intracisternal injection of the E protein. SARS-CoV-2 virus caused microglial activation and astrogliosis, according to a postmortem case study [49]. Microglia and astrocytes are CNS resident cells and play a crucial role in homeostasis and neuroinflammation [50]. We found that the inhibition of microglia, but not astrocytes, alleviated the E protein-induced depression-like behaviors and olfactory disorder. As the innate immune cells in the brain, microglia are more sensitive to pathogens than astrocytes and serve as the main mediators of neuroinflammation [51]. In response to CNS injury, microglial activation is essential for host defense and neuron survival [52]. However, persistent activation and dysregulation of microglia may result in deleterious and neurotoxic consequences by overproduction of a variety of cytotoxic factors such as TNF-α [53]. Toxins and pathological stimuli injure neurons, which is enhanced and amplified by overactive microglia [54]. This then further causes more extensive damage to nearby neurons and ultimately promotes pathogenic outcomes [55, 56].

On the other hand, both microglia and astrocytes can release pro-inflammatory mediators upon activation. In line with these findings, the E protein showed elevated expression of pro-inflammatory cytokines IL-1β and IL-6 in cortex, hippocampus, and olfactory bulb. Recent findings showed that increased levels of IL-1β and IL-6 were discovered in the cerebral fluid of patients with COVID-19 infection and neurological symptoms [57, 58]. Importantly, IL-1β and IL-6 have been identified as targets for alleviating the clinical condition, because they are thought to produce detrimental effects including the neurotoxic effect and inflammation. IL-1β has various biological activities. It increases neuronal apoptosis as well as neuronal loss by NMDA-evoked, glia-triggered, and/or proNGF-mediated pathways [59–62]. IL-6 also has been linked to neuronal cell death [63]. Furthermore, we observed that the E protein reduced the expression of ZO-1 in various brain regions. IL-1β and IL-6 have been shown to enhance blood–brain barrier permeability through cytokines-induced tight junction degradation, particularly ZO-1 and claudin-5 [64–66]. Alteration of the BBB integrity increases the opportunity for the viruses and cytokines to pass the BBB and facilitate the infiltration of periphery immune cells into the CNS, resulting in brain injury and exacerbating neuropsychiatric symptoms [67, 68].

The E protein may induce the production of inflammatory factors through two potential mechanisms. TLR2 can sense the E protein [18, 69]. The E protein specifically interacts with the TLR2 pathway, activating NF-κB and inducing the release of pro-inflammatory cytokines and inflammatory chemokines. Additionally, it was found that the SARS-CoV envelope protein interacted with syntenin of the host cell. This interaction caused syntenin to be redistributed to the cytoplasm, where it caused the upregulation of inflammatory cytokines [70]. This would cause an exacerbated immune response, leading to tissue damage, edema, and ultimately the characteristic acute respiratory distress syndrome, which was consistent with the histopathological characteristics induced by the SARS-CoV-2 E protein in the mouse spleen and lung [48].

TLRs identify pathogen-associated molecular patterns (PAMPs) and trigger immune responses on interaction with infectious pathogens. TLR1–9 are expressed in microglia. The expression of TLRs in microglia is controlled in response to pathogens [71]. Microglial activation and neurotoxicity have been connected to TLRs [72, 73]. Several TLRs (TLR1, TLR2, TLR4, etc.) have an association with disease progression in patients with COVID-19 [18]. In particular, TLR2, which can sense the E protein, is necessary for inflammatory responses to coronavirus. We also found that the E protein upregulated TLRs in multiple brain regions and triggered microglial inflammatory response via TLR2 in vivo. TLR2 is crucial for the microglia in response to viruses [74]. TLR2 activation induced microglia to release NO and other cytotoxic substances through multiple ligands [75].

This study has several limitations. We found that the E protein caused neuroinflammation by activating TLR2 and led to depression-like behaviors and dysosmia. Although C29 simultaneously inhibited the activation of microglia while suppressing TLR2, given that TLR2 receptors are expressed in both astrocytes and neurons, it would be a more explicit approach to use the Cre-loxP system to further knock out TLR2 in microglia. In the CNS, microglia constantly monitor the microenvironment and produce substances that have an impact on nearby astrocytes and neurons [76]. Although the interplay between glial cells and neurons is crucial in brain pathophysiology [77], it is impossible to disregard the immediate impact of SARS-CoV-2 on neurons. SARS-CoV-2 can infect neurons directly [78, 79]. Whether the E protein causes injury to neurons and there is a relationship between this effect and the neurological complications of SARS-CoV-2 deserve further exploration.

## Supplementary Information

**Additional file 1: Figure S1.** Inhibition of astrocytes did not alleviate depression-like behaviors and dysosmia induced by E protein. A. Representative images of GFAP expression in the cortex, hippocampus regions, and olfactory bulb from E protein-treated mice receiving Vehicle or LAA injection. B-C. Fluorescence area analysis showed that LAA significantly downregulated GFAP expression in the cortex, hippocampus, and olfactory bulb. D. The percentage of sucrose water consumption for PBS- or E protein-treated female and male mice receiving Vehicle or LAA injection. E. The immobility duration in TST test for PBS- or E protein-treated female and male mice receiving Vehicle or LAA injection. F. The immobility duration in FST test for PBS- or E protein-treated female and male mice receiving Vehicle or LAA injection. G. The latency time for discovering the sunflower seed in olfactory measurement in PBS- or E protein-treated female and male mice receiving Vehicle or LAA injection. n ≥ 6 per group; **p < 0.01, ***p< 0.001, ****p < 0.0001; n.s., no significance; Student’s t-test.

**Additional file 2: Figure S2.** Inhibition of microglia by Minocycline attenuated the expression of neuroinflammatory mediators induced by E protein. A-B. qRT-PCR analysis showed that intracisternal injection of Minocycline downregulated IL-1β, TNF-α, IL-6, CCL2, MMP2, and CSF1 in the cortex and hippocampus. C. qRT-PCR analysis showed that intracisternal injection of Minocycline downregulated IL-1β, IL-6, and CCL2 in the olfactory bulb. n = 4; **p < 0.01, ***p < 0.001, ****p < 0.0001; n.s., no significance; Student’s t-test.

**Additional file 3: Figure S3.** Blocking TLR2 by C29 attenuated the expression of neuroinflammatory mediators induced by E protein. A-B. qRT-PCR analysis showed that intracisternal injection of C29 downregulated IL-1β, TNF-α, IL-6, CCL2, MMP2, and CSF1 in the cortex and hippocampus. C. qRT-PCR analysis showed that intracisternal injection of C29 downregulated IL-1β, IL-6, and CCL2 in the olfactory bulb. n = 4; *p < 0.05, **p < 0.01, ***p < 0.001, ****p < 0.0001; n.s., no significance; Student’s t-test.

**Additional file 4: Table S1.** Target gene.

## Data Availability

There are no data, software, databases, and application/tools available apart from those reported in the present study. All data are provided in the manuscript and supplementary data section.

## Abbreviations

CNS: Central nervous system
E protein: SARS-CoV-2 envelope protein
SPT: Sucrose preference test
FST: Forced swimming test
TST: Tail suspension test
OM: Olfactory measurement
LAA: L-α-Aminoadipate
PAMPs: Pathogen-associated molecular patterns
TLR2: Toll-like receptor 2
TLRs: Toll-like receptors
