# Correction: SARS‑CoV‑2 envelope protein triggers depression‑like behaviors and dysosmia via TLR2‑mediated neuroinflammation in mice

**DOI:** 10.1186/s12974-026-04097-3

**Published:** 2026-11-01

**Authors:** Wenliang Su, Jiahang Ju, Minghui Gu, Xinrui Wang, Shaozhuang Liu, Jiawen Yu, Dongliang Mu

**Affiliations:** 1 Department of Anesthesiology, Peking University First Hospital, Beijing, China; 2 Liangzhu Laboratory, MOE Frontier Science Center for Brain Science and Brain‑Machine Integration, State Key Laboratory of Brain‑Machine Intelligence, Zhejiang University, Hangzhou, 311121 China; 3 Department of Rehabilitation Medicine, Tongji Hospital, Tongji Medical College, Huazhong University of Science and Technology, Wuhan, Hubei China; 4 Department of Pharmacy, Beijing Chaoyang Hospital, Capital Medical University, Beijing, China; 5 Department of Urology, Shengjing Hospital of China Medical University, Sanhao Street 36, Shenyang, Liaoning 110004 China; 6 Department of Anesthesiology, Peking Union Medical College Hospital, Chinese Academy of Medical Sciences and Peking Union Medical College, Beijing, China

**Correction: Journal of Neuroinflammation 20:110** **(2023)**

**https://doi.org/10.1186/s12974-023-02786-x**

Following the publication of this article [1], the author noticed the errors in Figures 7 and 10. During figure preparation, the IF data of IBA1 in Figure 7A (Male, DG, E+Veh group) and Figure 10A (Female, CA3, E+C29 group) were inadvertently misplaced which were identified during the examination of the original data. The updated figures are presented with this correction article.

**Fig. 7:**
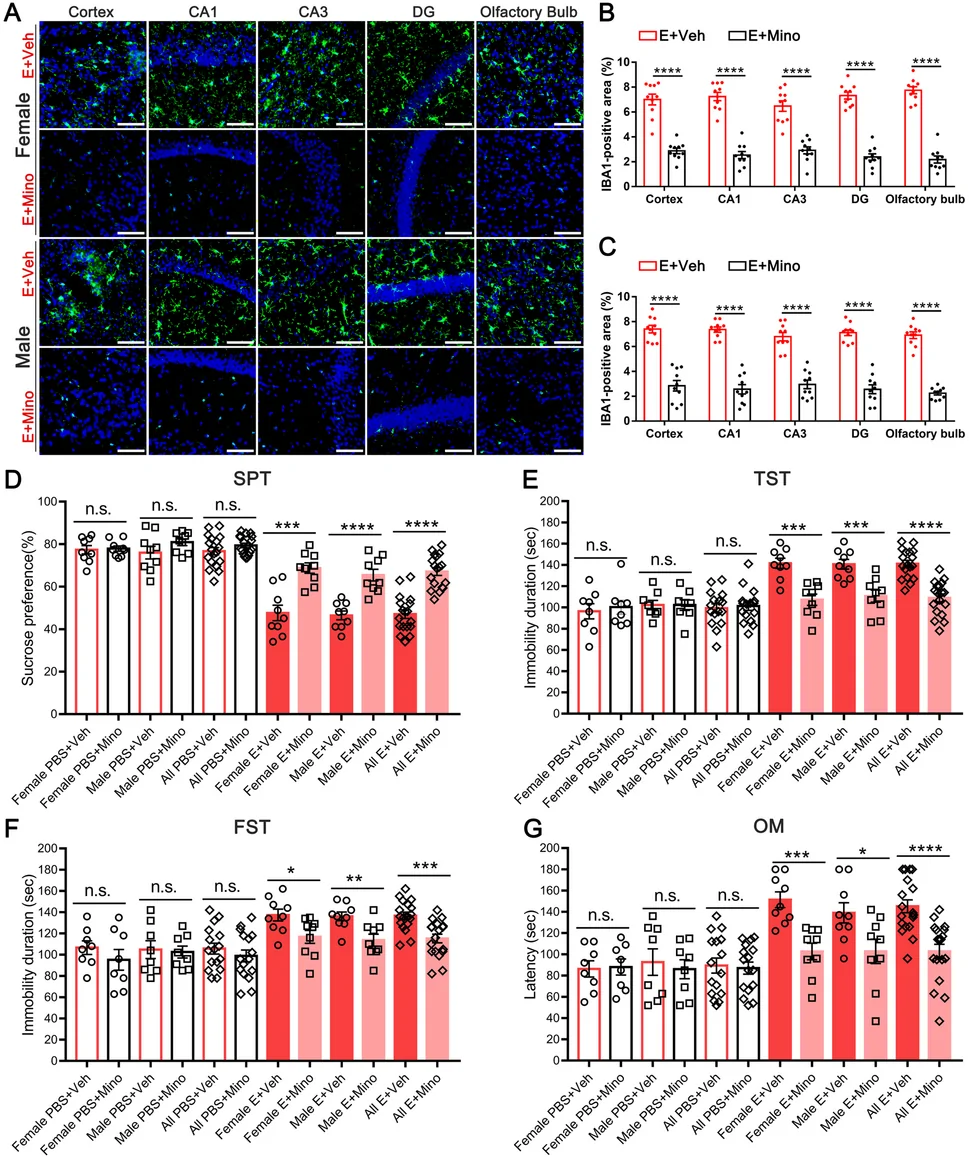
Inhibiting microglia alleviated depression-like behaviors and dysosmia induced by E protein. **A** Representative images of IBA1 expression in the cortex, hippocampus regions, and olfactory bulb from E protein-treated mice receiving Vehicle or minocycline injection. **B**, **C** Fluorescence area analysis indicated that minocycline significantly downregulated IBA1 expression in the cortex, hippocampus (CA1, CA3, and DG regions), and olfactory bulb (Scale bar: 50 μm). **D** The percentage of sucrose water consumption for PBS- or E protein-treated female and male mice receiving Vehicle or minocycline injection. **E** The immobility duration in TST test for PBS- or E protein-treated female and male mice receiving Vehicle or minocycline injection. **F** The immobility duration in FST test for PBS- or E protein-treated female and male mice receiving Vehicle or minocycline injection. **G** The latency time for discovering the sunflower seed in the olfactory measurement for PBS- or E protein-treated female and male mice receiving Vehicle or minocycline injection. n ≥ 6 mice per group; *p < 0.05, **p < 0.01, ***p < 0.001, ****p < 0.0001; n.s., no significance; Student’s t-test

**Fig. 10:**
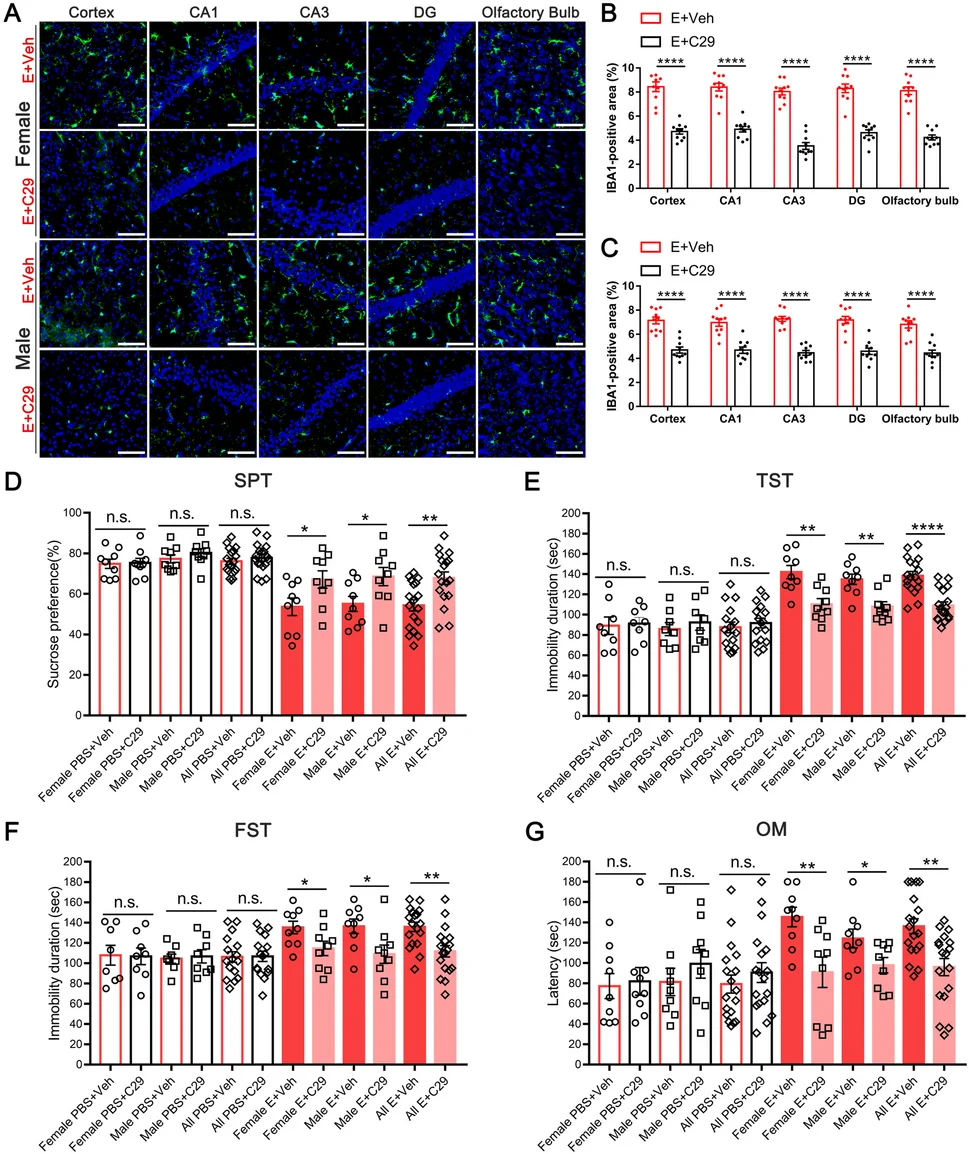
Blocking TLR2 alleviated depression-like behaviors and dysosmia induced by E protein. **A** Representative images of IBA1 expression in the cortex, hippocampus regions, and olfactory bulb from E protein-treated mice receiving Vehicle or C29 injection (scale bar: 50 μm). **B**, **C** Fluorescence area analysis showed that C29 significantly downregulated IBA1 expression in the cortex, hippocampus (CA1, CA3, and DG regions), and olfactory bulb. **D** The percentage of sucrose water consumption for PBS- or E protein-treated female and male mice receiving Vehicle or C29 injection. **E** The immobility duration in TST test for PBS- or E protein-treated female and male mice receiving Vehicle or C29 injection. **F** The immobility duration in FST test for PBS- or E protein-treated female and male mice receiving Vehicle or C29 injection. **G** The latency time for discovering the sunflower seed in olfactory measurement in PBS- or E protein-treated female and male mice receiving Vehicle or C29 injection. n ≥ 6 per group; *p < 0.05, **p < 0.01, ****p < 0.0001; n.s., no significance; Student’s t-test
