# Supplementary figures and images for: SARS-CoV-2 envelope protein triggers depression-like behaviors and dysosmia via TLR2-mediated neuroinflammation in mice
Source: J Neuroinflammation. 2023 May 8;20:110. doi: 10.1186/s12974-023-02786-x (PMC10166055; doi:10.1186/s12974-023-02786-x)

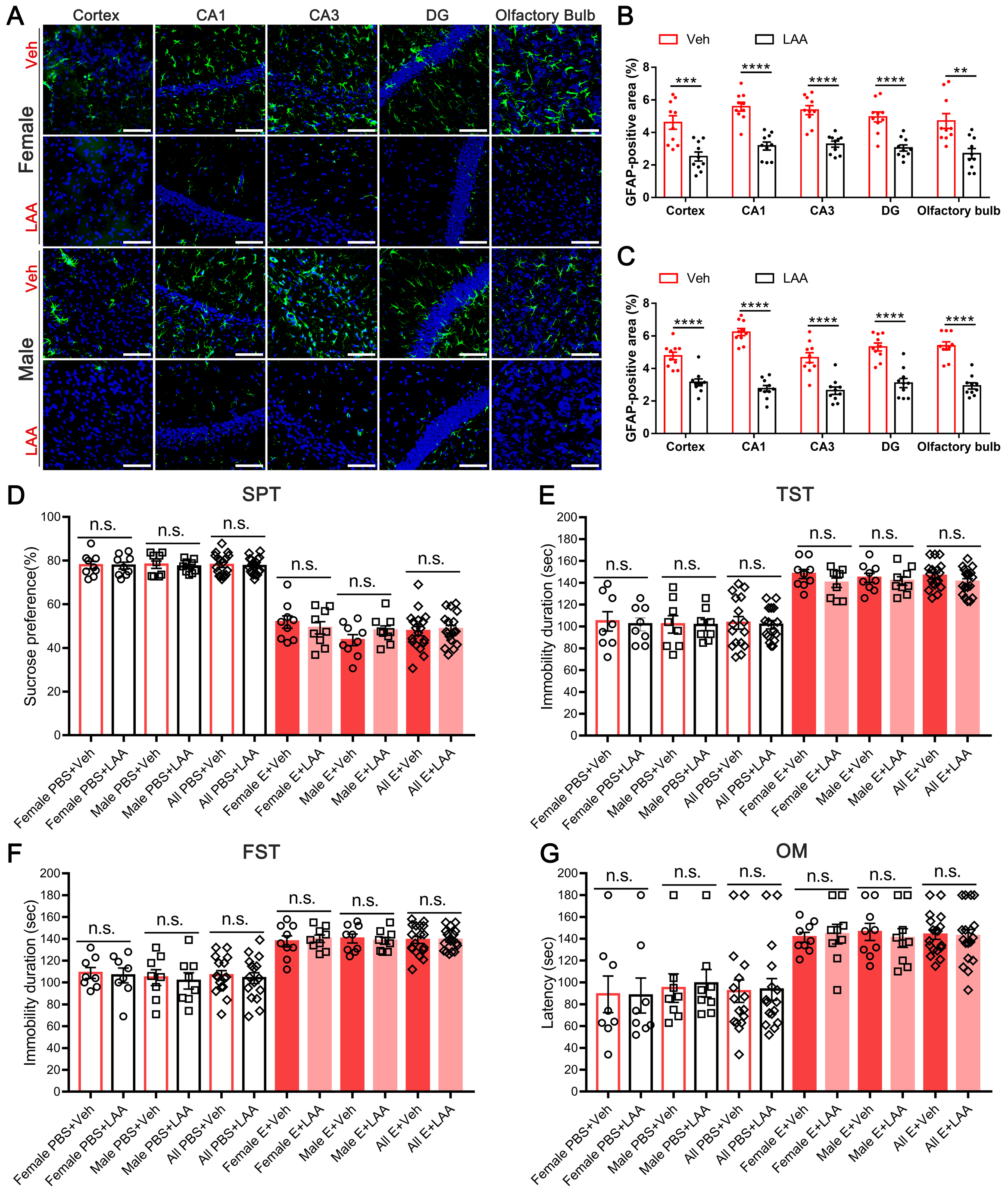

Supplement: Supplementary file 1 — Additional file 1: Figure S1. Inhibition of astrocytes did not alleviate depression-like behaviors and dysosmia induced by E protein. A. Representative images of GFAP expression in the cortex, hippocampus regions, and olfactory bulb from E protein-treated mice receiving Vehicle or LAA injection. B-C. Fluorescence area analysis showed that LAA significantly downregulated GFAP expression in the cortex, hippocampus, and olfactory bulb. D. The percentage of sucrose water consumption for PBS- or E protein-treated female and male mice receiving Vehicle or LAA injection. E. The immobility duration in TST test for PBS- or E protein-treated female and male mice receiving Vehicle or LAA injection. F. The immobility duration in FST test for PBS- or E protein-treated female and male mice receiving Vehicle or LAA injection. G. The latency time for discovering the sunflower seed in olfactory measurement in PBS- or E protein-treated female and male mice receiving Vehicle or LAA injection. n ≥ 6 per group; **p < 0.01, ***p< 0.001, ****p < 0.0001; n.s., no significance; Student’s t-test. [file 12974_2023_2786_MOESM1_ESM.jpg]

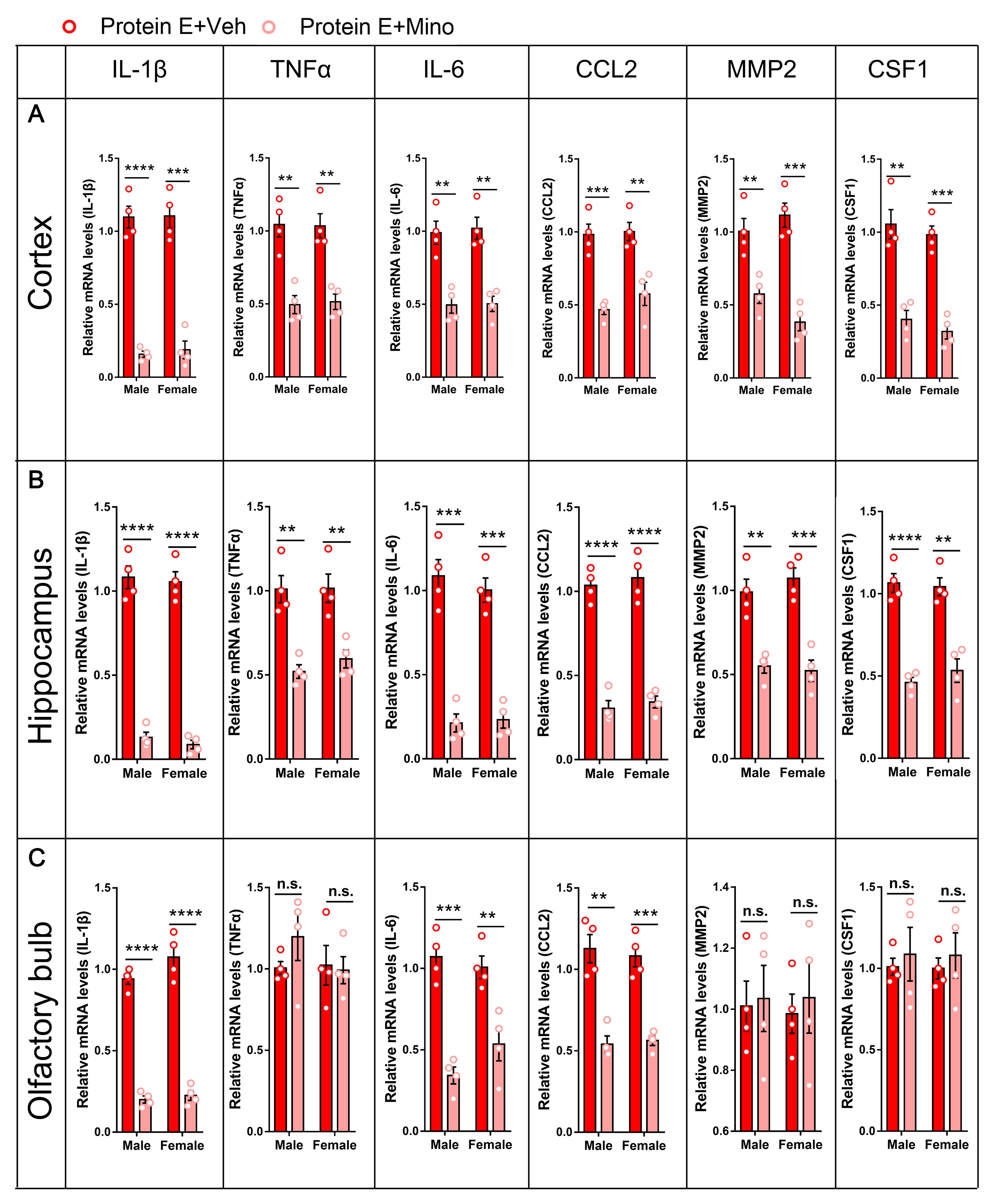

Supplement: Supplementary file 2 — Additional file 2: Figure S2. Inhibition of microglia by Minocycline attenuated the expression of neuroinflammatory mediators induced by E protein. A-B. qRT-PCR analysis showed that intracisternal injection of Minocycline downregulated IL-1β, TNF-α, IL-6, CCL2, MMP2, and CSF1 in the cortex and hippocampus. C. qRT-PCR analysis showed that intracisternal injection of Minocycline downregulated IL-1β, IL-6, and CCL2 in the olfactory bulb. n = 4; **p < 0.01, ***p < 0.001, ****p < 0.0001; n.s., no significance; Student’s t-test. [file 12974_2023_2786_MOESM2_ESM.jpg]

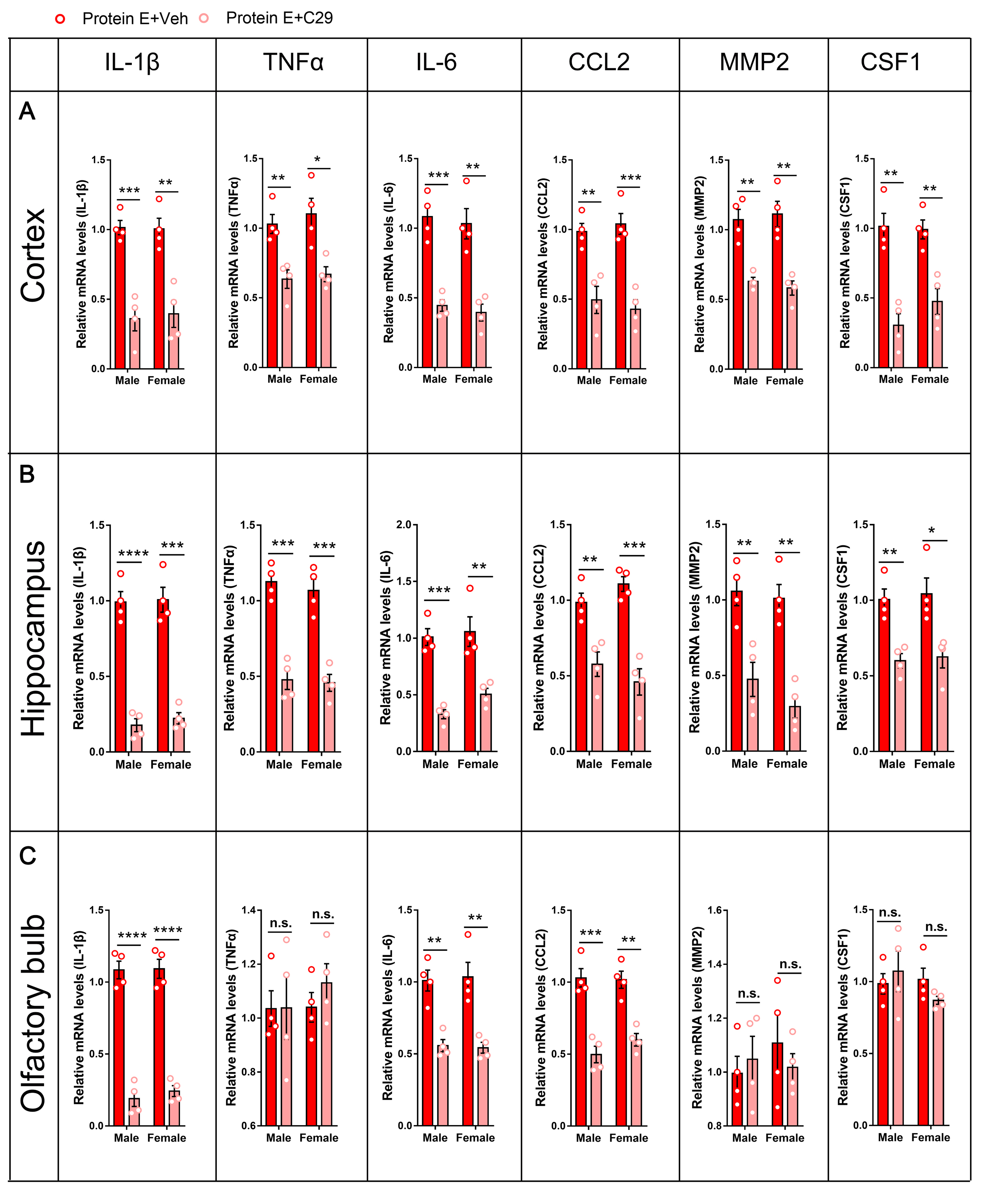

Supplement: Supplementary file 3 — Additional file 3: Figure S3. Blocking TLR2 by C29 attenuated the expression of neuroinflammatory mediators induced by E protein. A-B. qRT-PCR analysis showed that intracisternal injection of C29 downregulated IL-1β, TNF-α, IL-6, CCL2, MMP2, and CSF1 in the cortex and hippocampus. C. qRT-PCR analysis showed that intracisternal injection of C29 downregulated IL-1β, IL-6, and CCL2 in the olfactory bulb. n = 4; *p < 0.05, **p < 0.01, ***p < 0.001, ****p < 0.0001; n.s., no significance; Student’s t-test. [file 12974_2023_2786_MOESM3_ESM.jpg]
